# Probing the potential of rare earth elements in the development of new anticancer drugs: single molecule studies

**DOI:** 10.3762/bjnano.16.15

**Published:** 2025-02-14

**Authors:** Josiane A D Batista, Rayane M de Oliveira, Carlos H M Lima, Milton L Lana Júnior, Virgílio C dos Anjos, Maria J V Bell, Márcio S Rocha

**Affiliations:** 1 Departamento de Física, Universidade Federal de Juiz de Fora, Juiz de Fora, Minas Gerais, Brazil; 2 Departamento de Física, Universidade Federal de Viçosa, Viçosa, Minas Gerais, Brazil; 3 Departamento de Ciências Naturais, Universidade Federal do Acre, Rio Branco, Acre, Brazil

**Keywords:** DNA, optical tweezers, rare earth elements, single molecule force spectroscopy

## Abstract

We use optical tweezers and atomic force microscopy to investigate the potential of rare earth elements to be used as anticancer agents in the development of new chemotherapeutic drugs by characterizing the binding of three rare earths (ytterbium, neodymium, and erbium) to double-stranded DNA, which is one of the main targets for these drugs inside cells. The three elements presented a significant interaction with the biopolymer in buffers of physiological relevance, typically binding with very high equilibrium association constants (10^6^ to 10^7^ M^−1^) at the DNA grooves. Furthermore, neodymium and erbium can also induce a very strong compaction/condensation of the double helix at high concentrations, promoting DNA collapse at the single molecule level in a similar way to what occurs with classical DNA condensing agents such as polycations and depletants.

## Introduction

The development of new drugs to treat human diseases is a field of singular importance that usually involves interdisciplinary research to find, produce, and test drug candidates until they can reach the market [1–2].

Cancer chemotherapy, for instance, is a type of treatment that deserves improvements not only in the efficacy of the drugs employed to kill tumor cells, but also in reducing the occurrence of the well-known side effects related to these therapies. Actually, both aspects depend on the development of new drugs and/or drug carriers that can improve the selectivity of these anticancer agents to reach their specific targets inside tumor cells [3–5]. Although commonly used in a number of technological applications, rare earth elements are yet unexplored in the development of new drugs for cancer chemotherapies, and only a few works have pointed out the potential of such elements for this field [6–8].

An initial motivation to investigate the potential of rare earth elements for cancer treatments is the fact that some metals have been successfully used, or are being investigated, as components of chemotherapeutic drugs [9–10], especially platinum [11–16] or, alternatively, ruthenium [17], titanium [18], gold, and copper. Here we report the high potential of three rare earth elements (ytterbium, neodymium, and erbium) to interact with double-stranded DNA (dsDNA) in buffers of physiological relevance. This is an important issue since dsDNA is one of the main targets of anticancer drugs inside cells; hence, a compound that interacts significantly with the biopolymer presents an interesting potential to be used in the development of novel chemotherapeutic drugs.

The results found here show that, in general, rare earth elements are promising agents to be used in the development of new anticancer drugs, presenting high binding equilibrium constants with the double helix structure. Furthermore, depending on the concentration used, two of the rare earths (erbium and neodymium) tested also present the ability to compact/condense DNA, which opens the door for other types of applications such as gene therapies and the design of drug carriers themselves. To achieve such results, we performed single-molecule force spectroscopy using optical tweezers (OT) on DNA complexes formed with the three rare earths at various concentrations. The mechanical properties of these complexes were then determined as a function of the element concentration. From these data, the physical chemistry of the interaction was extracted as well, providing robust information about the effects of the rare earths on the DNA double helix [19,16]. In addition, atomic force microscopy (AFM) imaging assays were also performed to confirm DNA compaction/condensation by erbium and neodymium, allowing for a direct visualization of these condensates and therefore complementing the OT study. These results point out that rare earth elements should be considered for further research studies in DNA science.

## Experimental

### Optical tweezers assays

Rare earth oxides (X_2_O_3_, X = Yb, Nd, or Er) were bought from Sigma-Aldrich and used without further purification. Concentrate rare earth stock solutions (≈1 mM) were prepared by dissolving each oxide in deionized water, slowly adding a small quantity of HCl since these oxides are insoluble in water. From these stocks, less concentrated solutions were prepared at various rare earth concentrations by diluting the stock solutions in a phosphate-buffered saline (PBS) buffer with [Na^+^] = 150 mM, pH 7.4.

The samples used for OT assays consist of biotin-labeled λ DNA molecules (New England Biolabs N3011S) tethered by the ends between a streptavidin-coated coverslip and a streptavidin-coated polystyrene bead (3 μm diameter), mounted in a custom-made sample chamber where the surrounding PBS buffer can be exchanged. The experiment starts by stretching a bare DNA molecule, obtaining its characteristic force–extension curve (FEC), which is fitted to the Marko–Siggia worm-like chain (WLC) model [20] to determine the two main mechanical parameters in the entropic regime, that is, the contour and persistence lengths of the DNA molecule. To guarantee the accuracy of the results, the chosen DNA is stretched using only low forces (*<*5 pN); this type of measurement is repeated six times, obtaining the average values of the mechanical parameters and their error bars (standard error of the mean) [21].

After this characterization, the chosen rare earth is introduced in the sample chamber at the desired concentration, and the procedure described above is repeated using the same DNA molecule, thus obtaining the average values of the mechanical parameters for the complexes formed with a fixed rare earth concentration in the sample. With this procedure, the graphs of the two mechanical parameters as functions of the compound concentration are constructed for the three different rare earths used here. This procedure has been proved to be very robust in determining changes in the mechanical properties of DNA–ligand complexes as a function of the ligand concentration in the sample. The complete details can be found in [21].

### A model to determine the binding parameters from the persistence length data

A quenched-disorder statistical model that describes DNA interactions with small ligands was developed by our group in the past [22,19]. Such a model allows one to extract the binding parameters of a given interaction from the data of the persistence length as a function of the ligand concentration in the sample [22].

In summary, for DNA ligands that induce monotonic changes on the persistence length upon binding, the effective measured value (*A*_E_) of this mechanical parameter can be written as


[1]

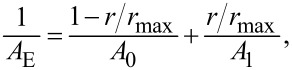



where *A*_0_ is the persistence length of the bare DNA molecule, *A*_1_ is the local persistence length induced by the ligand upon binding on a site (or, equivalently, the persistence length at bound ligand saturation), *r* is the bound site fraction (fraction of DNA base pairs occupied by bound ligand molecules), and *r*_max_ is the saturation value of *r* [19].

Equation 1 can be related to the binding parameters of the interaction by using a binding isotherm that captures the physical chemistry of such interaction via the parameter *r*. A well-known binding isotherm is the Hill model, which is the simplest isotherm that accounts for cooperativity in binding reactions [19],


[2] $$\frac{r}{r_{\max}}=\frac{{\left(KC_{\text{f}}\right)}^{n}}{1+{\left(KC_{\text{f}}\right)}^{n}},$$

where *C*_f_ is the free (not bound to DNA) ligand concentration, *K* is the equilibrium association binding constant, and *n* is the Hill exponent, a parameter that measures the cooperativity degree of binding reactions. If *n >* 1, the interaction is positively cooperative, that is, a bound ligand molecule increases the effective affinity of DNA for subsequent ligand binding. If *n <* 1, the interaction is negatively cooperative, and a bound ligand molecule decreases the effective affinity of DNA for subsequent ligand binding. If *n* = 1, the interaction is non-cooperative, and the effective affinity is independent on the number of bound ligand molecules.

The binding parameters and the local persistence lengths are left as adjustable parameters to be determined from the fit. The details of this methodology can be found in [19,21].

### Atomic force microscopy assays

The samples for atomic force microscopy (AFM) assays consist of 3 kbp DNA molecules (ThermoFischer Scientific SM1711) in 10 mM Tris-HCl buffer with the addition of 10 mM of MgCl_2_ (pH 8.0), which is needed to reliably deposit the DNA molecules on mica substrates. PBS buffer cannot be used here since its high NaCl concentration disturbs the DNA adsorption on the substrates. The solution of DNA + ligand is first prepared in a microtube and allowed to equilibrate for ca. 30 min. Then, an aliquot of 20 μL is deposited on the substrate and completely dried, first with nitrogen at ambient temperature (≈25 °C) and then in a fridge (4 °C) for 12 h.

The 3 kbp DNA was used here to allow for the visualization of various distinct molecules in the scanned images and to avoid relevant volume exclusion effects that play a significant role for λ DNA because of its larger contour length (48.5 kbp) [23].

The mica substrates were scanned with the AFM operating in the tapping mode. All experiments were performed in air, at ambient temperature and with a humidity between 20% to 30%. This experimental procedure has been proved suitable to visualize deposited DNA and DNA–ligand complexes in a reproducible and reliable way [23–24].

The AFM instrument used in the experiments was a NT-MDT-NTEGRA PRIMA operating in tapping mode with TAP300 Al-G tips (Budget Sensors).

## Results and Discussion

### Force spectroscopy

**Figure 1:**
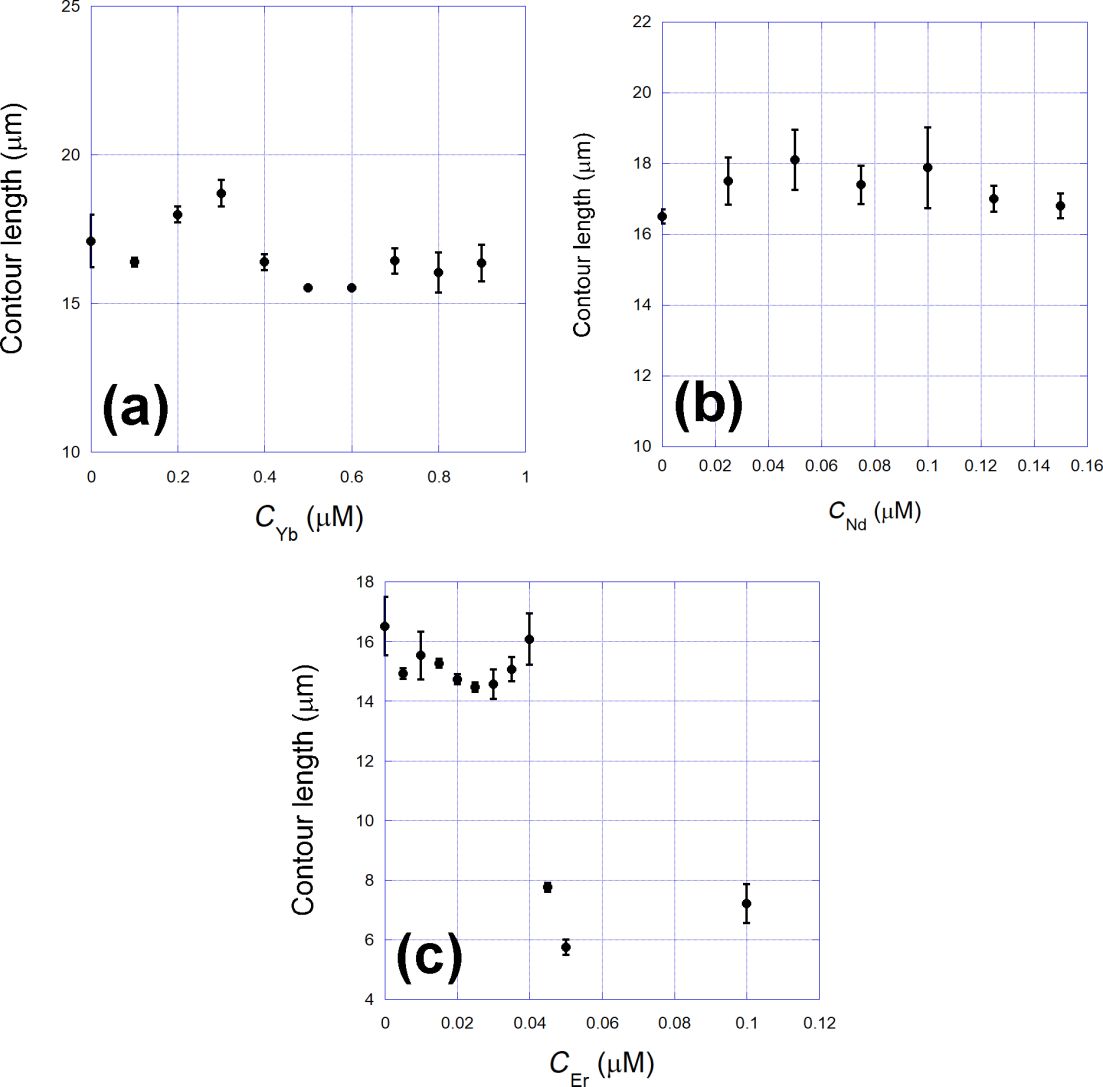
Contour length measured as a function of the ligand concentration for the three types of complexes formed between DNA and the rare earths. (a) Ytterbium, (b) neodymium, and (c) erbium. For ytterbium and neodymium, this mechanical parameter remains constant. In the case of erbium, the behavior is different. The contour length remains initially constant until reaching a certain threshold concentration (≈0.04 μM) and then decays abruptly, indicating that a strong DNA compaction process has occurred. A similar compaction was also found for neodymium at higher concentrations (*>*0.5 μM).

In Figure 1 we show the contour length measured as a function of the ligand concentration for the three types of complexes formed between DNA and the rare earths. For ytterbium and neodymium, this mechanical parameter remains constant at the concentration range shown, indicating that a binding reaction, if it occurs, does not change the average interspace between consecutive base pairs. In the case of erbium, in contrast, the behavior is completely different. The contour length remains initially constant until reaching a certain threshold concentration (≈0.04 μM) and then decays abruptly, indicating that a strong DNA compaction process has occurred [25–28]. A similar DNA compaction was also found for neodymium at higher concentrations (*>*0.5 μM), but not for ytterbium.

**Figure 2:**
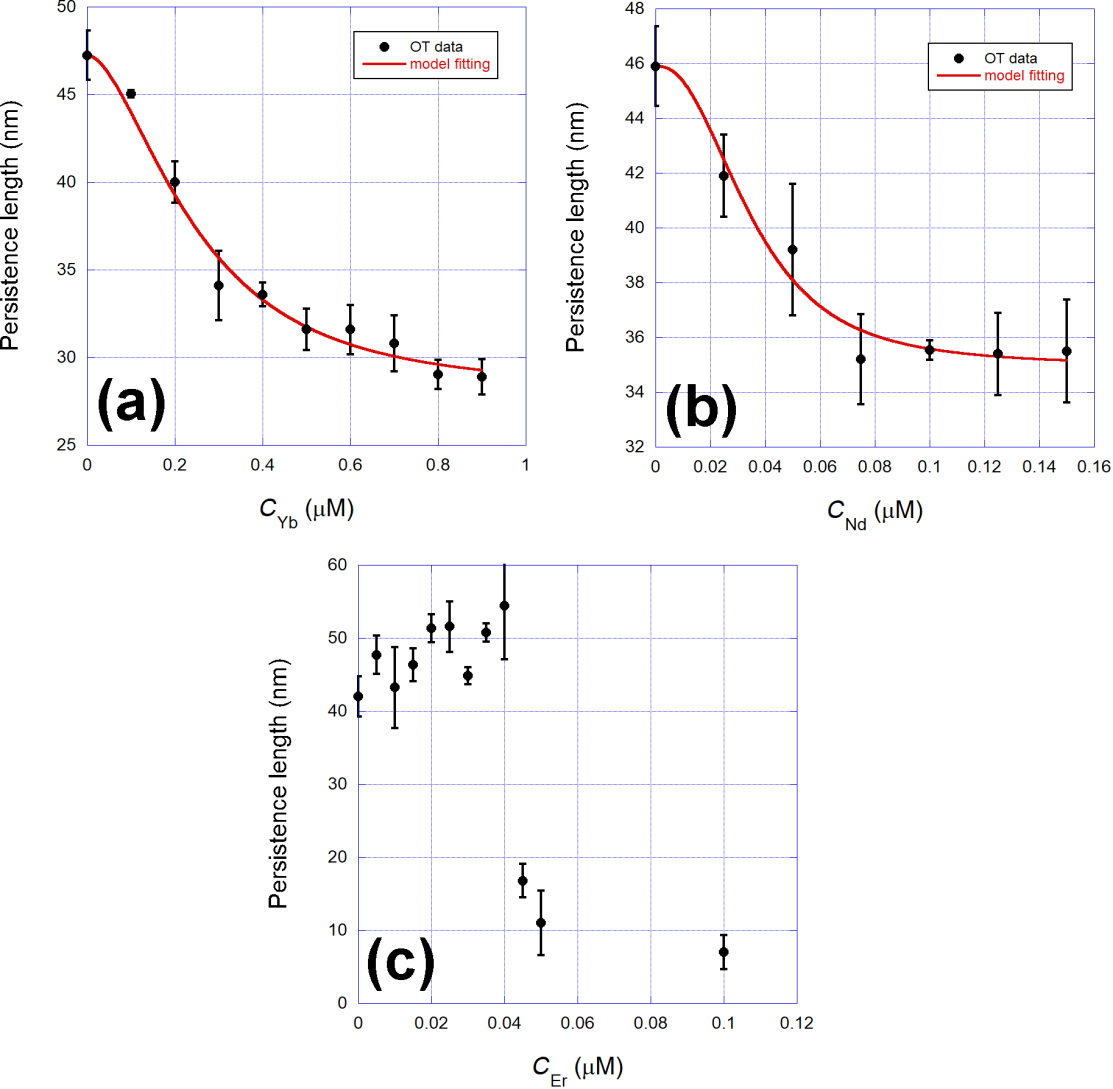
Persistence length of the three types of complexes studied. (a) Ytterbium, (b) neodymium, and (c) erbium. The cases of ytterbium and neodymium are similar, with the persistence length presenting a monotonic decrease as a function of the ligand concentration. The case of erbium is again different, with the persistence length presenting an initial very slight increase and then an abrupt decrease at the same concentration where the contour length also presented a similar abrupt decay (≈0.04 μM). Solid lines: fit to the persistence length quenched-disorder statistical model from which the binding parameters can be determined.

In Figure 2, we show the corresponding persistence length of the three types of complexes studied. Observe again that the cases of ytterbium and neodymium are similar, with the persistence length presenting a monotonic decrease as a function of the ligand concentration. This behavior confirms that there is an interaction between these two rare earths and the DNA double helix. Although such interaction does not induce any change of the contour length of the complexes formed at low rare earth concentrations, it induces effective bends leaving the double helix more flexible with respect to the bending rigidity, which is reflected in the decrease measured for the effective persistence length. The case of erbium is again different, with the persistence length presenting an initial very slight increase and then an abrupt decrease at the same concentration where the contour length also presented a similar abrupt decay (≈0.04 μM).

The behavior of the mechanical properties of the DNA complexes formed with the ytterbium and neodymium is very similar to the one previously studied using europium [7]. In this work, we showed that europium binds outside the double helix in a cooperative way, forming clusters of about approx. three molecules and presenting an equilibrium association constant of the order of 10^5^ M^−1^, that is, a considerably strong binding. To advance in the comparison, we fit the persistence length data of the complexes formed between DNA and ytterbium and neodymium with the quenched-disorder model presented, which is the same model used to analyze the corresponding europium data in [7]. The fits are also shown in Figure 2 as solid lines and allowed us to determine the relevant binding parameters of the interactions, which are schematically shown in Table 1.

**Table 1:** Binding parameters and local persistence length of the DNA complexes with Yb and Nd, determined from model fits. *K* is the equilibrium association binding constant, *n* is the Hill exponent, and *A*_1_ is the saturation persistence length.

Rare earth	*K* (M^−1^)	*n*	*A*_1_ (nm)

Yb	(3.7 ± 0.8) × 10^6^	1.9 ± 0.3	28 ± 2
Nd	(2.9 ± 0.6) × 10^7^	2.6 ± 0.5	35 ± 2

The equilibrium association binding constant of the interactions between Yb and Nd with DNA are of the order of 10^6^ and 10^7^ M^−1^, respectively. These binding constants are even higher than that found for Eu, that is, one and two orders of magnitude higher, respectively. Such results suggest that Yb and Nd bind very strongly to the double helix and, thus, reinforce the idea that rare earth elements are good candidates for the development of drugs that have DNA as their target inside cells [7], for example, chemotherapeutic drugs. In addition, the Hill exponent *n* obtained for the two elements suggests that they bind in a positive cooperative way forming clusters of about two to three molecules, a situation similar to that found for Eu. Finally, the values found for the local persistence length *A*_1_ reflect the fact that the interactions induce a decrease on the effective persistence length of the complexes formed, probably by promoting bends at the binding sites.

For the DNA complexes formed with erbium, in contrast, both mechanical parameters behave completely differently, as previously shown in Figure 1c and Figure 2c, suggesting a strong DNA compaction for compound concentrations above 0.04 μM. Such discontinuous behavior cannot be fitted with our quenched-disorder model to determine the binding parameters with accuracy. Nevertheless, observing the relevant concentration range in which the interaction occurs (i.e., below 0.1 μM), it is evident that the interaction is very strong and should occur with an equilibrium constant as high as in the cases of ytterbium and neodymium. Furthermore, it is worth to mention that a small ligand that presents such a strong ability to compact DNA can easily find applications in fields such as drug delivery and gene therapy.

### Atomic force microscopy

**Figure 3:**
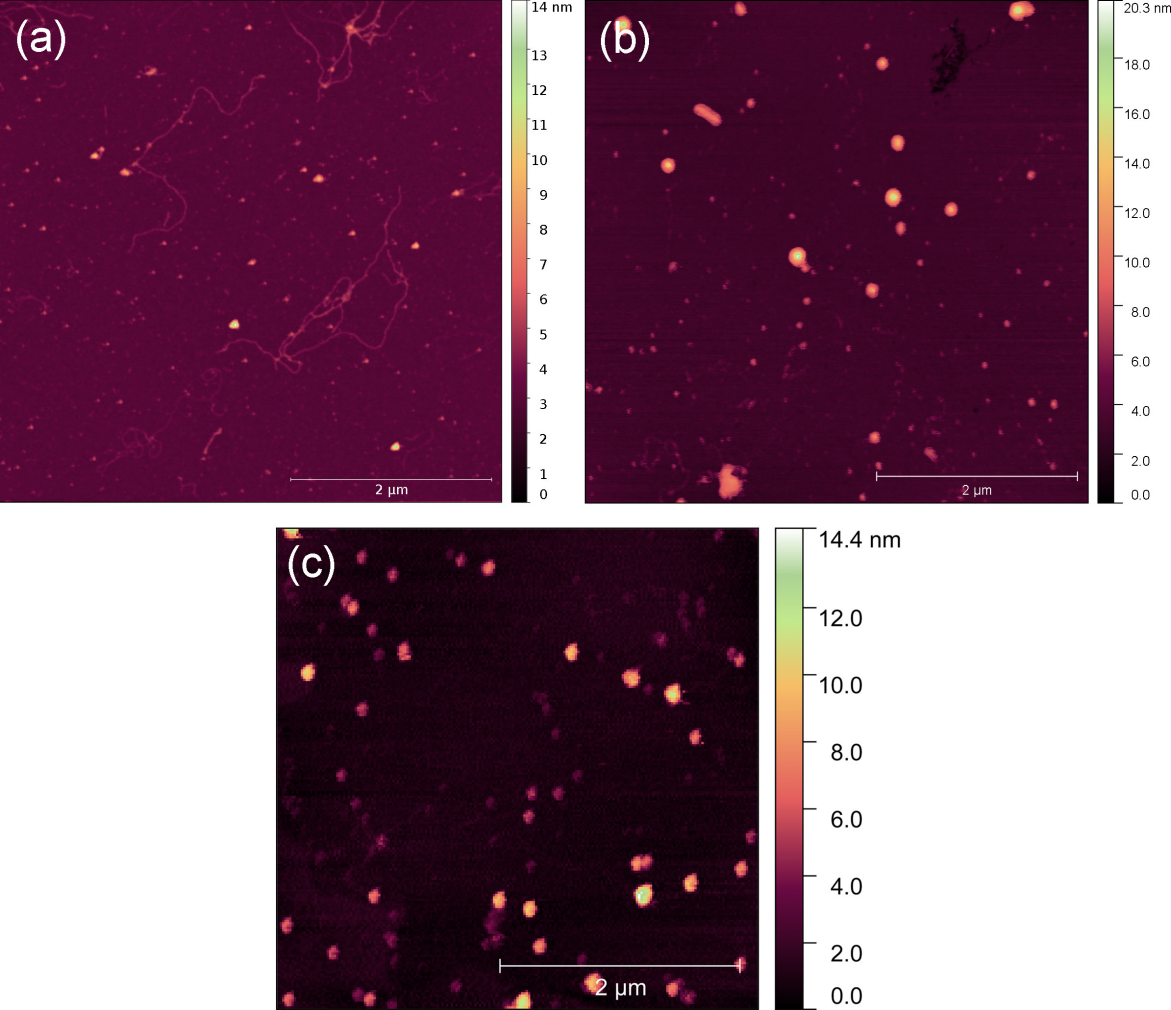
Typical images of the complexes formed between DNA and the rare earths obtained from our AFM assays. (a) Ytterbium, (b) neodymium, and (c) erbium.

In Figure 3 we show typical images of the complexes formed between DNA and the rare earths obtained from our AFM assays. Without any rare earth in the sample, the deposited DNA molecules exhibit the usual 2D worm-like chain morphology [23]. The concentration of rare earths used in each case was 1 μM, which is sufficiently high to able DNA compaction in the present cases (if such compaction actually occurs for the rare earth used).

Observe that for ytterbium (Figure 3a), the DNA molecules deposited on the mica substrates are not compacted/condensed, in agreement to what was concluded from the OT experiments. For neodymium (Figure 3b), the complexes appear compacted/condensed, which is also in agreement with the OT results since the concentration used in the sample to obtain this image was 1 μM of neodymium. Finally, for erbium (Figure 3c) the complexes also appears compacted/condensed for 1 μM of the rare earth, also in agreement with the OT results.

We stress that the intent of the AFM experiments performed here was to confirm the ability of neodymium and erbium to condense DNA at high concentrations (*>*0.5 μM for neodymium and *>*0.05 μM for erbium). Furthermore, these assays allowed us to estimate the typical shape and size of these condensates. They present a globular morphology with typical heights around 20 nm, which is compatible to results found for DNA condensates formed with well-known condensing agents [29].

Finally, it is worth to discuss the mechanism of DNA condensation by the rare earths verified here both by optical tweezers and AFM. The most likely explanation for such condensation is related to the use of HCl to dissolve the rare earth oxides in solution during the sample preparation process (see section “Experimental”). It is well known that these oxides react with aqueous HCl producing the trivalent cation form of the rare earth elements and water [30]. Thus, the trivalent rare earth cations can interact with the double helix, promoting the well-known cation-induced DNA condensation process, just as other polycations such as, for example, spermidine and hexaammine cobalt [29,31]. Note that the threshold concentration for DNA condensation to occur depends strongly on the specific rare earth element, as discussed in the above paragraph. Thus, although condensation by ytterbium was not observed here, it could possibly occur at higher concentrations not assessed in the present work.

## Conclusion

We investigated the interaction of three rare earth elements (ytterbium, neodymium, and erbium) with double-stranded DNA molecules. They exhibited a significant interaction with the biopolymer, binding with very high equilibrium association constants (10^6^ to 10^7^ M^−1^) at the DNA grooves. In addition, it was verified that neodymium and erbium can also induce DNA condensation at high concentrations. These results suggest that rare earth elements can be used in the design of new drugs that have DNA as their target inside cells (e.g., chemotherapeutic drugs) or in the development of new carriers for drug delivery systems because of their ability to condense DNA. Therefore, this work points out that rare earth elements should be considered for further research studies in DNA science.

## Data Availability

Data generated and analyzed during this study is available from the corresponding author upon reasonable request.
